# The Influence of Data Clustering on the Generalization Properties of Neural Networks Using the Example of Determining the Geographical Origin of Wine

**DOI:** 10.3390/foods15142511

**Published:** 2026-07-16

**Authors:** Aleksan Khalafyan, Zaual Temerdashev, Aleksey Abakumov, Vera Akinshina

**Affiliations:** Analytical Chemistry Department, Faculty of Chemistry and High Technologies, Kuban State University, 149 Stavropolskaya Street, Krasnodar 350040, Russia; statlab@kubsu.ru (A.K.); temza@kubsu.ru (Z.T.); ak-vera@yandex.ru (V.A.)

**Keywords:** classification, clustering, neural network, performance, generalizing properties, scatterplots, canonical values, clustering metrics

## Abstract

Based on the elemental composition of 359 samples of dry wines (*Riesling*, *Chardonnay*, *Muscat*, *Cabernet Sauvignon*, and *Merlot*) produced in the Krasnodar Territory (Russia), the influence of data clustering on the generalizing properties of neural network models for solving classification problems was studied. Clustering as a property of predicted classes being compact and separate from each other was evaluated using scatterplots of canonical values from discriminant analysis and the Silhouette Score, Calinski-Harabasz, Davies-Bouldin, and Dunn metrics. With a decrease in data clustering, the generalization properties of neural network models decrease despite an increase in dataset size. Since the predictive properties of neural networks are primarily correlated with the clustering of data, it seems logical to say that their growth will occur when “quantity turns into quality”, that is, clustering will increase with increasing dataset size. The validity of the assumption explains the polarity of trends observed in literature. With the growth of training datasets, some researchers record improvements in the predictive properties of models, while others record deterioration. The results obtained are of great practical importance, as they indicate the unsuitability of unlimited data accumulation and allow optimizing the costs of collecting it, which is important, especially for food quality control.

## 1. Introduction

Contrary to current trends in the development and implementation of artificial intelligence (AI), in science, various applied fields, medicine, pharmaceuticals, and the development of new materials, as well as the assessment of food quality, including wines, there are currently no theoretically sound and structured rules for designing and training artificial neural networks (NN) [1]. According to V. Betelin [2], one of the reasons for the poor applicability of NN-based systems is a lack of theoretical justification for their stability and convergence, which guarantees a reliable result. AI system developers are trying to improve NN-based technologies by designing new architectures, learning algorithms, and types of networks [3], or resorting to heuristic rules whose effectiveness is not substantiated and is highly questionable. Questions about the capabilities of neural networks, their architecture, rules for selecting activation functions, etc., are relevant from the point of view of reliability and are actively discussed in the global scientific community. There has been a significant increase in the number of research studies in which authors associate the reliability of NNs with the quality of data. They assume that the quality of a dataset is associated with its size a priori. It is presumed that the larger the dataset, the more reliable it is and, consequently, the better the learning and generalization properties of a neural network [4,5]. The authors of [4] use a dataset of students enrolled in professional training programs to explore the widespread belief that machine learning models perform better when they have a larger training dataset. The research did not confirm this point of view. It showed that with an increase in observations, the accuracy and performance of the model increased slightly, and the significance of the data was determined by its quality, rather than just its quantity. In [5], the authors describe the problem of the discrepancy between the large number of features and the small number of observations when designing new materials using deep learning, which usually leads to poor performance. To solve the problem, it is proposed to use a reduction in the number of features, as well as data augmentation. It shows that the balance between the number of observations, features, or model parameters should attract more attention when managing the dataset size.

Many heuristic rules try to regulate the ratio of the number of columns of data (features) to the number of rows (observations)—1:10, 1:50, etc. The authors of [6] investigated the impact of the quality of training datasets on the performance, accuracy, and complexity of machine learning models. It is shown that datasets may contain errors or inconsistencies that can occur during collection, aggregation, or description. It can lead to inaccurate analytics and unreliable decisions. Researchers are more focused on improving the quality of neural network models, but efforts to improve data quality are insufficient. Evaluating data quality using special metrics and developing appropriate methods to eliminate data deficiencies will allow developers to reduce the time needed for iterative debugging of machine learning models in order to improve their performance. Incomplete, erroneous, or unsuitable training data can lead to the creation of unreliable models on which incorrect decisions can be based. Thus, the development of reliable applications based on artificial intelligence requires high-quality training and testing of data that is characterized by accuracy, completeness, and consistency [7].

The authors of [8] summarized the results of the study on the concept of data quality in machine learning, including the definition of related concepts, analysis of quality issues and risks, and quality parameters and indicators throughout the life cycle of the dataset. Certain efforts in the pharmaceutical industry focus on developing new drugs using deep learning algorithms. This includes changes in data representation, the study of various characteristics to more accurately describe the structure of proteins, ligands, and their interactions with or without three-dimensional complex structures, as well as variations in NN architectures [9]. However, the improvement in forecasting results may be related not only to improvements in algorithms but also to the quality and quantity of data. The impact of these factors on the predictive properties of convolutional neural networks (CNNs) has not been sufficiently studied. When studying the effect of dataset size on prediction accuracy, subsets of different sizes are randomly selected. This is followed by a comparison of the constructed deep learning models on modified datasets. The diverse benefits of AI are being realized in drug development, including increased performance, accuracy, and risk minimization [10]. It has been shown that AI accelerates the search for potential drugs by predicting pharmacokinetics, toxicity, and possible side effects, as well as improving the design of clinical trials through improved patient recruitment and data analysis. The authors of [10] discuss not only the diverse advantages of AI in drug development but also critical issues such as data quality, the interpretability of models, and regulatory barriers. The problem of assessing the effect of data quality on NN properties is considered in [11]. It analyzes methods for evaluating and optimizing the quality of datasets in the context of object detection tasks using images, using Vendi Score (VS) and its improved version, Quality-Weighted Vendi Scores. VS and qVS metrics evaluate the diversity and quality of datasets. When all *n* objects are diverse, then VS = *n*. If all objects are identical, then VS = 1. Their research shows that models trained on subsets optimized for diversity using qVS exhibit improved performance. Diverse subsets result in higher accuracy and generalization capabilities compared to randomly selected or less diverse datasets of the same size.

An analysis of the works mentioned above indicates the importance and relevance of recent studies on the impact of data quality on the reliability of NN models being developed. Although in some of these studies, data quality is equated with its quantity. At the same time, we note that there are virtually no studies among them that examine the quality of data in terms of its structural features, in particular, the presence of clustering when solving classification problems. Clustering refers to the compactness and separation of predicted classes, assuming similarity (uniformity) of objects within and difference (heterogeneity) between classes. Weakening of data clustering refers to a reduction in a model’s capacity to form distinct, meaningful, and well-separated data groupings, often caused by high dimensionality, algorithm mismatch, or noise. This phenomenon leads to lower discriminative power and reduced interpretability, requiring mitigation techniques such as dimensionality reduction or robust scaling. In [12], the influence of clustering on the generalization properties of neural network models was investigated.

The authors of [13,14,15,16,17,18,19] solved the applied problem of classifying the geographical origin of wines using neural networks. During this process, they noted the influence of clustering data on the elemental composition of wine on its prognostic properties. In this research, this task serves as a model example for investigating a fundamental issue: the dependence of the predictive properties of a neural network on the degree of clustering of data. Thus, the emphasis is shifted from the applied result to a general methodological conclusion. The purpose of this study is to investigate the influence of cluster data structures on the change in prognostic properties of NN, which is illustrated by considering the problem of establishing the geographical origin of wine. For this purpose, data on the elemental composition of 359 dry wine samples was used, on the basis of which 7 data sets (subsets) were formed with decreasing clusterization.

Clusterization was assessed visually and using numerical clustering metrics: Silhouette Score [20], Calinski-Harabasz [21], Davies-Bouldin [22], and Dunn Index [23]. The VS and qVS metrics are not analogous to the above clustering metrics, which characterize the compactness and separability of data. However, they are a fairly powerful and flexible tool for evaluating the overall diversity of data.

## 2. Materials and Methods

### 2.1. Objects of Research

The role of clustering in generalizing the properties of NNs was studied by analyzing the elemental composition of 359 samples of dry white (Riesling, Chardonnay, and *Muscat*) and red (*Cabernet* and *Merlot*) wines produced in the main geographical areas of the wineries of the Krasnodar Territory. CJSC “Zaporozhskoye”, LLC “Kuban-Vino”, OJSC APF “Fanagoria”, LLC APC “Milstream-Black Sea Wines”, CJSC AF “Kavkaz”, CJSC “Abrau-Durso”, CJSC APC “Gelendzhik”, CJSC AF “Myskhako”, LLC “Firma Sommelier”, LLC AF “Sauk-Dere”, LLC “Soyuz-Vino”. Wine producers belong to different geographical zones (subzones) of the Krasnodar Territory: South foothill (72 samples), Taman (210 samples), and Black Sea (77 samples) zones [16]. The choice of a specific dataset is due to several interrelated reasons. First and foremost, the data was obtained in the context of specific analytical studies and reflects the actual elemental composition of wine. The use of real data is crucial for verifying the generalizing properties of neural networks under conditions of natural variation in the chemical composition of products. Secondly, the long-term scientific background of the author’s team in chemometric analysis of wine quality enables them to correctly interpret the results, reasonably assess the quality of the model, and eliminate issues related to wine production technology in enterprises. Wine samples were provided by manufacturers or purchased from stores. The provided samples were bottled in dark green glass bottles with screw caps and stored at 10 °C. According to the manufacturer, the content of ethanol in the wines was 9–13%, and the titratable acidity was 4–7 g/L. Information about the wines studied is given in Supplementary Table S1.

### 2.2. Elemental Analysis

The concentrations of 15 elements (Al, Ba, Ca, Cu, Fe, K, Li, Mg, Mn, Na, Ni, Rb, Sr, Ti, and Zn) in wines were determined using the ICP-AES method (iCAP 7400 Thermo Scientific, Waltham, MA, USA). The calibration solutions were obtained using single-element standard solutions with a concentration of 1000 mg/L (Inorganic Ventures, Christiansburg, VA, USA). Calibration curves of microelements (Al, Ba, Cu, Fe, Li, Mn, Ni, Rb, Sr, Ti, and Zn) were in the concentration range from 1 to 1000 µ/L. When determining macroelements (Ca, K, Mg, and Na), calibration curves were in the concentration range of 50–10,000 µ/L. The solutions were prepared using deionized water with a maximum resistivity of 18.2 MΩ·cm^−1^, which was obtained using the DuoPUR (Milestone, Milan, Italy) sub-distillation system. The preparation of wines for analysis was carried out by diluting 15 times (for determination of microelements) and 100 (for determination of macroelements) with 2% nitric acid, taking into account literature data [16,24].

### 2.3. Statistical Analysis

The STATISTICA 13.3 (Tibco, Palo Alto, CA, USA) package is used as a data analysis tool [24]. In the package environment, basic statistics of the concentrations of 15 elements were calculated for wine samples; canonical discriminant analysis was used to construct scatterplots to visualize the cluster structure of wine production regions. NN models were built to predict the geographical origin of wines; correlation coefficients and scattering diagrams were calculated to assess the relationship between clustering metrics, dataset size, and the generalizing abilities of NNs. To calculate the metric values, we used silhouette_score, calinski_harabasz_score; davies_bouldin_score dunn functions from the sklearn.metrics module of the scikit-learn Python(version 1.9.0) library.

### 2.4. Datasets Modeling

According to the content of the elements in wines, 7 sets (subsets) were formed, with consistently decreasing clustering and increasing the size of the datasets. The concentrations of 15 elements in wine samples were used as predictors in the classification model, and three regions of grape growth were predictive classes. To model the subsets, class centers are defined as points in a 15-dimensional space, with coordinates equal to the average concentrations of elements in wine samples taken from the Black Sea, Taman, and Southern Foothill zones in amounts of 210, 77, and 72, respectively. Table 1 shows the average ($\bar{x}$), minimum (min), maximum (max) concentration values, and standard deviations (s).

Subsets 1, 2, 3, 4 and 5 include samples of wines from three regions, with boundaries spaced from the centers at distances equal to 1·s; 1.5·s; 2·s; 2.5·s; 3·s. Therefore, the bounds of metal concentrations for the sets are calculated using formulas:

(1) $$\mathrm{left}\;\mathrm{bound}\;=\bar{x}-k\cdot s$$

(2) $$\mathrm{right}\;\mathrm{bound}=\bar{x}+k\cdot s$$

where k = 1.0; 1.5; 2.0; 2.5; 3.0.

Table 2, Table 3 and Table 4 show the ranges of metal concentrations for all 5 subsets of each region, with negative values for the left bounds set at 0.

To develop the “evidence-based” part of the research, 5 sets of initial data (subsets) were expanded to 7. In our opinion, this made it possible to assess the dynamics of the decrease in the generalizing properties of NN depending on the weakening of data clustering. Subset 6 represents the initial dataset, whose lower and upper bounds correspond to the minimum and maximum concentrations of the elements (Table 1). In subset 7, the wine samples from the original dataset were randomly distributed between regions while maintaining their quantitative representation. The composition of wine samples from different regions for subsets 1–7 is shown in Table 5.

## 3. Results and Discussion

### 3.1. Analysis of the Cluster Structure of Wine Samples Elemental Composition Datasets

**Figure 1:**
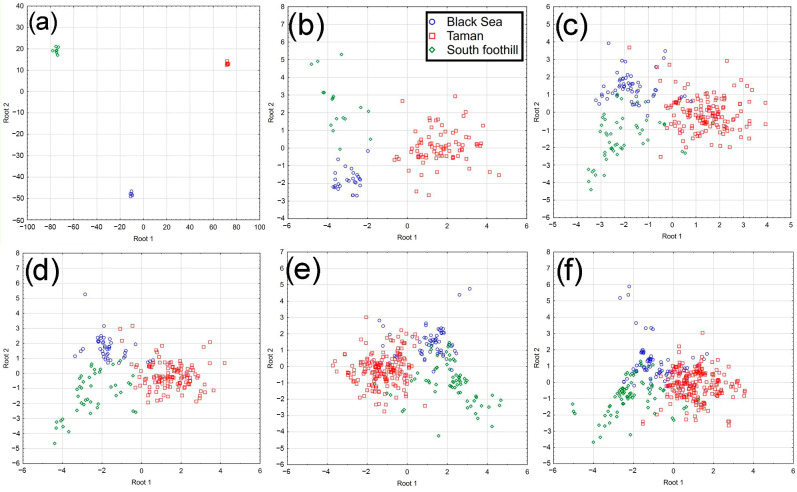
Scatterplots of canonical values for (**a**) subset 1; (**b**) subset 2; (**c**) subset 3; (**d**) subset 4; (**e**) subset 5; (**f**) subset 6.

**Figure 2:**
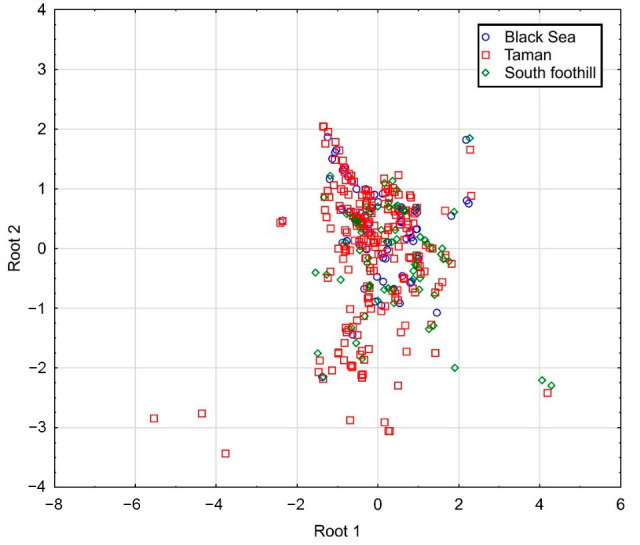
Scatterplots of canonical values for subset 7.

The dynamics of changes in the bounds of the subsets (Table 2, Table 3 and Table 4) indicate a consistent increase in the variations in concentrations of elements from different regions, which is a prerequisite for a decrease in class uniformity and weakening of the data clustering. The tabular representation of the data does not reflect the cluster structure of the regions well, so we will use scatterplots of canonical values from discriminant analysis (Figure 1 and Figure 2).

Scatterplots make it possible to transfer wine samples, which are objects in a space of dimension 15, to a space with a smaller dimension—a plane in the canonical coordinate system (root 1 and root 2) while maintaining the order of distances between them. The number of canonical roots, also called discriminant functions, is equal to the number of classes minus 1, or the number of predictors of the model minus 1, whichever is smaller. Preserving the order of distances means that objects that are close/distant in a multidimensional space will also be close/distant (similar/different) on a plane. Therefore, scatterplots, as an alternative to PCA, allow you to visually assess the clustering structure, i.e., to qualitatively evaluate the clustering of data.

The distances between classes in discriminant analysis are determined by the squared Mahalanobis distance. It shows the distance between class centers and serves as an objective assessment of the cluster structure. From Figure 1a, it can be seen that the samples within the regions of subset 1 are close, and the samples from different regions are apart. Thus, subset 1 has a clear cluster structure with pronounced clustering features: compactness within classes and separation between objects of different classes. Distances Black Sea/Taman, Black Sea/South foothill, and Taman/South foothill for subset 1 are 10,571, 8669, and 21,701, respectively. As can be seen from Figure 1b and the distances for subsets 2 (25, 18, 33), the clustering in subset 2 has become significantly less than that in subset 1. Nevertheless, the bounds between the classes remain, and there are no examples of wines in “foreign” territories. A slightly different pattern was observed for subsets 3–6. As the number of observations in the datasets increase, the spread of element concentrations increases, and clustering decreases significantly. The distances between the zones for subsets 3 to 6 are significantly smaller compared to sets 1 and 2 and are equal to 13, 10, and 17 (subset 3); 11, 9, and 12 (subset 4); 8, 7, and 12 (subset 5); and 6, 6, and 11 (subset 6). The random distribution of regions between the wine samples, as was done with the set of 7, led to one homogeneous population without a cluster structure (Figure 2). The distances between the zones were 0.22, 0.33, and 0.30, which are greatly less compared to the subsets. 1–6. There are no signs of clustering (compactness and separation) in subset 7. Thus, Figure 1 and the distances between the centers of the regions indicate that subsets 1–7 were constructed with a consistent decrease in clustering from its maximum severity to its complete absence.

An additional confirmation of the conclusions based on graphical information is the dynamics of changes in clustering metrics as the number of clusters increases. Metrics quantify various aspects of clustering. They are based on different mathematical formulas and, in some cases, may contradict each other. Therefore, their combined use is recommended [20,21,22,23].

The Silhouette Score (S-S) metric is calculated for a single object using the equation:

(3) $$\text{S-S}=\frac{b-a}{\max\left\{a,b\right\}}$$

where: a—the average distance from an object to all other objects in the same cluster.; b—the average distance from it to all objects in its nearest neighboring class, for which b has the minimum value. The resulting value of the metric is the average value of S for the entire dataset. S-S evaluates how close an object is to its class compared to its neighbors. It takes values from −1 to 1: clusters are very dense and well separated. 0—clusters overlap. Less than 0—objects belonging to classes may be erroneous.

The Calinski-Harabasz (C-H) metric is defined as the ratio of intercluster to intracluster dispersion normalized by the number of degrees of freedom:

(4) $$\text{C-H}=\frac{{SS}_{B}}{{SS}_{W}}\times \frac{N-k}{K-1}$$

where: N—the total number of objects in the sample; k—the number of classes; SS_B_—the sum of squared distances between clusters.; SS_W_—total intracluster variance (sum of squared distances within classes). The range of metric values is [0, +∞). High value indicates dense, well-separated clusters. The Davies-Bouldin (D-B) metric [22] is calculated using the equation:

(5) $$\text{D-B}=\frac{1}{k}\sum_{i=1}^{k}\underset{i\neq j}{max}\left(\frac{s_{i}+s_{j}}{d(c_{i},c_{j})}\right)$$

where: k—the number of classes, s_i_ and s_j_ are the average distances from the points in a class to its centroid (centroid); d(c_i_, c_j_)—the distance between centroids c_i_ and c_j_ of the i and j classes. Under the sum sign, a cluster j is selected for each class i, which maximizes the ratio (s_i_ + s_j_)/d(c_i_, c_j_). The Davis-Boldin metric measures the average “similarity” between classes: the distance between them is compared to their size. The range of metric values is between 0 and +∞, with smaller values being better for clustering.

The Dunn metric [23] is defined as the ratio of the minimum pairwise distance between points from different classes to their maximum diameter:

(6) $$\mathrm{Dunn}=\frac{\underset{1\leq i<j\leq m}{\min}d_{min}\left(C_{i},C_{j}\right)}{\underset{1\leq k\leq m}{\max}\Delta k}$$

where: m—the number of classes; d_min_ (*C*_i_, *C*_j_)—The distance between the nearest points of the *i* and *j* classes; Δk—diameter of the class k, the maximum distance between two points in the class. The range of metric values is [0, +∞). The larger it is, the higher the clustering of the data.

As can be seen from Table 6, the S-S, C-H, and Dunn mostly decrease with increasing subset size, while the D-B metric increases, which confirms the decrease in data clustering. Minor differences in the behavior of metrics are explained by different ways of estimating the data clustering and some similarity in the cluster structures of subsets 4–6 (Figure 1d–f). Of particular interest are the metric values for set 7, in which the cluster structure is completely absent. In this case, the S-S, C-H, and Dunn take values close to 0, and the value of D-B differs significantly from 0.

The presented results of the data clustering study show that canonical analysis and metric analysis complement each other and substantiate the claim of a consistent decrease in data clustering of subsets 1–7.

### 3.2. Modeling Predictive Neural Networks

The neural network models were built using 7 datasets using the Automated Neural Networks module of the STATISTICA package [25]. The program automatically divided the subsets into training and test datasets, using the sensor-based random number generator, in a ratio of approximately 70% to 30%. The number of wine samples in the training and test data of subsets 1–7 were 14 and 6 (subset 1); 87 and 36 (subset 2); 148 and 60 (subset 3); 180 and 76 (subset 4); 217 and 89 (subset 5); 252 and 107 (subset 6); and 252 and 107 (subset 7). Automated Neural Networks provide a program with an independent selection of optimal network parameters to minimize errors. These include the number of hidden layer neurons, an iterative learning algorithm, an error function, and activation functions for both hidden and output neurons. In order to ensure the correctness of the study, a total of 20 networks were built for each set under identical “default” program installation conditions. Of the 5 “best” networks selected by the program, we selected one with the highest predictive properties in the test dataset. These properties are characterized by network performance—the proportion of correctly classified objects. Performance on the training dataset determines the quality of NN training, while performance on the test dataset determines its generalizing abilities.

The NN parameters with the best generalizing properties are given in Table 7. In the designation of the NN architecture, the abbreviation MLP means a multilayer perceptron. The first number after “MLP” indicates the number of input neurons, which is equal to the number of predictors in the model. The second and third numbers indicate the numbers of hidden and output neurons in the model. The number of hidden neurons, as noted, is automatically selected by the program. The number of output neurons equals the number of predicted classes (in our case, there are 3 regions). Analysis of the data in the table shows that there is a consistent decrease in performance of models from subset 1 to set 7 in the test data: 100%, 100%, 98.08%, 97.37%, 97.37%, 93.25%, and 3.37%. It can be seen that when moving from subset 1 to 6, the generalizing properties of networks decrease gradually. At the same time, from subset 6 to 7, the accuracy of the forecast decreased by more than 27 times when the sizes of the sets were equal. At the same time, the performance of NN on the training data for subset 7 is quite high—91.85%. The explanation of the revealed phenomenon is quite simple. In the training data, the NN successfully adjusts the information at the output of the network (predicted classes) to the information at the input (initial classes). At the same time, due to the lack of clustering in subset 7, it is not possible to generalize the relationships built during training on the test sample, because they are not universal with respect to the entire dataset.

As can be seen from Table 7, weakening the data clustering leads to a decrease in model performance on the test data, despite an increase in the dataset size. For subset 1, which has the most pronounced cluster structure, the performance on the test data reached 100% using 15 predictors in the model. The model was trained on 14 samples and correctly classified 6 wine samples from the test data. At the same time, the number of predictors (15) exceeded the number of observations in the training data (14). However, a clearly defined cluster structure ensured maximum accuracy of the model. The wine samples from each region are located in their own specific areas of multidimensional space, which are located at significant distances from each other. For subset 7, where there is no cluster structure, the performance is only 3.37%, with a significantly larger number of observations (359) and the same number of predictors (15).

The results described above were obtained by modeling 7 subsets with a weakening cluster structure, in which dataset size increased. Next, we analyzed the behavior of predictive properties of NNs as dataset sizes increased that are not related to the modeling of cluster structure. Since, in the initial dataset, the number of wine samples from the Taman, Black Sea, and South foothills amounts to 210, 77, and 72, respectively (Table 5). At stage 1, the dataset is formed from the first 36 samples in approximately the same regional ratio of 3:1:1. Then, at stage 2, another 36 are added in the same proportion. This process continues until stage 10, when the dataset has reached 359 samples. The results of constructing the neural network for 10 samples, presented in Table 8, indicate that increasing the dataset size from 36 to 359 does not improve the generalizing properties of the models. In fact, it reduces them. The first four stages with a number of samples of 36, 72, 108, and 144 have the maximum possible performance of 100%. The study of the structure of constructed samples showed that a decrease in the generalizing properties of networks is also associated with clustering, which mainly decreases with increasing dataset sizes. The dynamics of changes in clustering metrics are shown in Table 9. It shows that the S-S, C-H, and Dunn metrics decrease steadily, while the D-B metric increases sharply. All four metrics clearly indicate a trend towards decreasing clustering with increasing dataset size.

The dynamics of changes in clustering metrics are shown in Table 9. It shows that S-S and C-H metrics decrease steadily, while the D-B metric increases gradually. All 4 metrics indicate a clear trend towards decreasing clustering with increasing dataset size.

**Figure 3:**
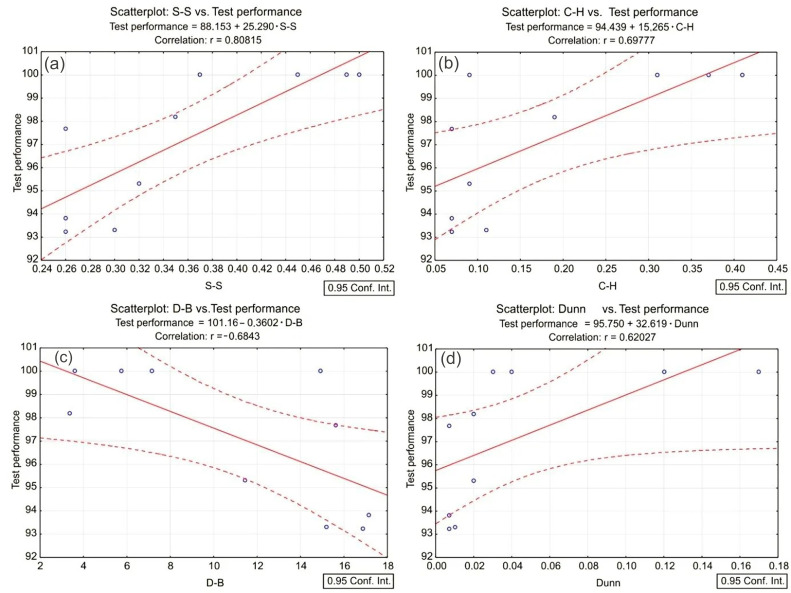
Scatterplot of the correlation between clustering metrics (**a**) S-S, (**b**) C-H, (**c**) D-B, and (**d**) Dunn and test performance.

**Figure 4:**
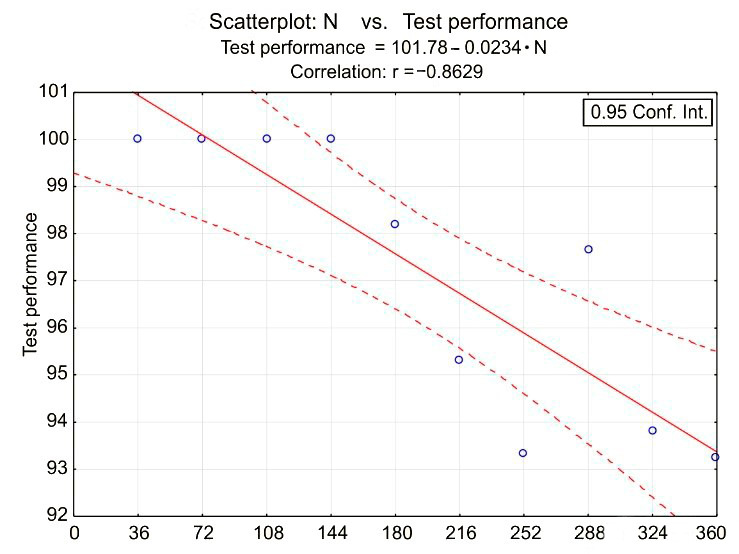
Scatterplot of the correlation between dataset size and test performance.

The patterns found in the influence of the sizes of 10 datasets on metrics and metrics on the generalizing properties of NNs are confirmed by moderate and strong correlations between metrics, dataset size, and test performance. This is determined using the Pearson’s correlation coefficient (Figure 3 and Figure 4). The regression lines in the scatterplots in Figure 3a–d illustrate the relationship between metrics and test performance. As the S-S, C-H, and Dunn metrics grow and the D-B metrics decrease, i.e., clustering increases, test dataset performance increases. The regression line in Figure 3 shows that an increase in sample sizes, reducing the values of the metrics S-S (r = −0.928), C-H (r = −0.648), and Dunn (r = −0.813) and, conversely, increasing the metric D-B (r = 0.728), leads to a decrease in the generalizing properties of NN.

**Figure 5:**
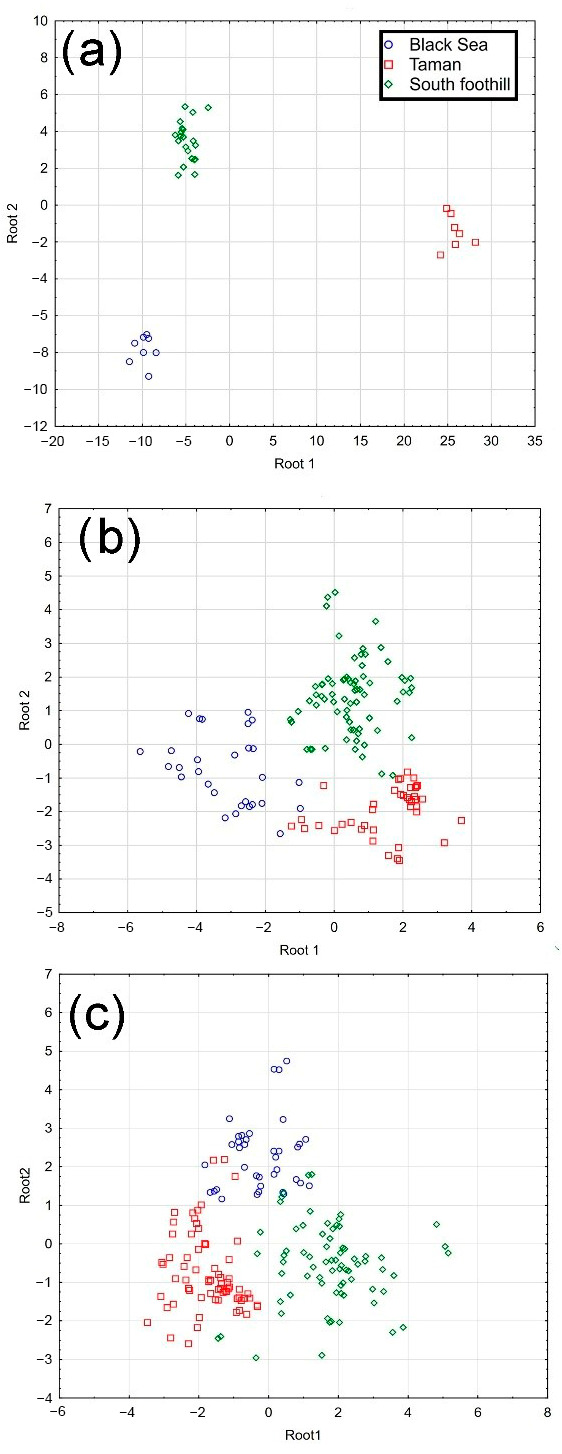
Scatterplot of canonical values for (**a**) 36 wine samples, (**b**) 144 wine samples, and (**c**) 180 wine samples.

The first four NN models presented in Table 8 correspond to a 100% test performance, despite the fact that dataset sizes are increasing: 36, 72, 108, and 144. Thus, data clustering decreases as the dataset size increases but remains high enough to achieve 100% test performance. This is well illustrated by the scatterplots (Figure 5a,b) for datasets with 36 (Stage 1) and 144 (Stage 4) observations. It can be seen that, despite the lower severity of clustering, the dataset of 144 wine samples still lacks samples of “friends among strangers” and “strangers among their own”, which are, as a rule, sources of erroneous classifications.

A slightly different picture is observed in the scatterplot for 180 wine samples (stage 5). Starting from stage 5, the generalizing properties of NN do not reach 100%. Figure 5c shows that the conditions of compactness and separation were not met. The samples from the South Foothill Zone ended up in the territory of two other zones, and the samples from Taman were among the Black Sea samples.

Practical experience shows that, with an increase in the number of constructed NN (n), the predictive properties of the model in the test data slightly increase, or at least do not decrease, approaching a certain threshold value. For the subset of 6 for = 20, 100, 500, 2500, and 5000, the test performance was compiled in a non-decreasing sequence: 93.25%, 94.38%, 94.38%, 95.51%, and 95.51%. From this, it can be concluded that a 250-fold increase in the number of NN results in a performance increase of only 2.26%. At the same time, the program optimized NN parameters, minimizing classification errors. Therefore, it is ineffective to further increase the number of NNs built and unlikely to exceed the performance threshold of 96% and, moreover, achieve error-free classification. This is explained by the fact that, in subset 6 (Figure 1f), there is a considerable number of objects that are homogeneous with objects from other classes, i.e., wine samples with similar concentrations of elements to samples from other regions. These samples are the source of misclassification.

The result of the presented studies leads to the conclusion that the objective factor determining the predictive properties of NNs in solving classification problems is not their architecture, learning algorithms, or parameters, but rather the quality of the data determined by its clustering.

## 4. Conclusions

The effect of clustering on the generalizing properties of neural network models was studied using subsets modeled from real datasets of the elemental composition of 359 wine samples, which included concentrations of 15 metals and wines from different geographical zones (Black Sea, Taman, and South Foothill). Clustering, implying the compactness and separation of predicted classes, was assessed using graphical analysis of the scatterplots of canonical values from discriminant analysis and numerical clustering metrics: Silhouette Score, Calinski-Harabasz Index, Davies-Bouldin Index, Dunn Index. Both approaches, based on different concepts, successfully complement each other. It is shown that, with a decrease of clustering, the performance of models on test data decreases, i.e., their generalizing properties. In the case of the highest clustering, the maximum possible test performance was 100% for a set with 15 predictors and 20 observations. However, in the absence of clustering, test performance was only 3.37% for the same set of 15 variables but for a larger set of observations (359).

The conducted studies have not confirmed the validity of the “myth” about the increase in predictive properties of neural networks with the increasing size of data. On the contrary, they decrease due to a decrease in clustering. It is probably fair to assume that an increase in the amount of data in classification tasks leads to an increase in generalizing properties of networks in conditions where “quantity turns into quality”, i.e., clustering increases. Otherwise, the generalizing properties of networks decrease, and further increases in data sizes become meaningless.

Thus, using the example of the problem of determining the geographical origin of wines, we show the influence of data clustering on the generalization properties of NN in the classification of objects. It was established that the higher the level of clustering, the better the predictive properties of neural networks. Obviously, the conclusions can be generalized to other machine learning methods, such as classification trees, support vector machines, k-nearest neighbors, and others.

## Figures and Tables

**Table 1:** Descriptive statistics of metal concentrations (µg/L) in wine samples (considering dilution) from the studied zones.

Element (Predictor)	Grape Growing Regions (Predictive Classes)											
	Taman (*N* = 210; *n* = 3)				Black Sea (*N* = 72; *n* = 3)				South Foothill (*N* = 72; *n* = 3)			
	$\bar{x}$	Min	Max	s	$\bar{x}$	Min	Max	s	$\bar{x}$	Min	Max	s
Al	986	168	2683	498	1581	347	8735	1551	1137	186	2261	541
Ba	136	35	291	55	122	47	241	47	181	50	459	91
Ca	65,670	19,410	111,077	15,626	66,538	37,162	91,254	11,927	63,498	39,304	99,074	15,209
Cu	172	<LOQ	5059	607	200	29	1249	257	56	<LOQ	159	30
Fe	3346	468	7928	1698	3737	514	8055	2056	3827	1265	6634	1283
K	735,951	300,800	1,496,000	237,661	803,860	195,612	1,422,800	345,948	959,442	354,717	1,851,000	333,096
Li	28	<LOQ	79	12	22	<LOQ	78	17	14	<LOQ	32	7
Mg	120,089	24,827	275,300	47,245	77,925	37,323	153,200	24,878	91,778	40,972	158,800	30,308
Mn	1373	95	3962	538	1173	580	1981	344	1102	366	2367	523
Na	50,515	8088	134,337	25,139	46,087	15,010	106,094	26,398	41,365	7915	102,120	21,691
Ni	100	<LOQ	3132	335	45	<LOQ	184	38	56	<LOQ	741	98
Rb	2041	109	9005	2210	808	278	2352	340	1040	204	4597	949
Sr	1051	411	2053	375	1127	489	3216	487	947	341	1938	479
Ti	14	<LOQ	50	11	16	<LOQ	51	11	25	<LOQ	65	14
Zn	597	<LOQ	7734	727	665	289	1457	245	614	213	997	211

**Table 2:** Bounds of data on element concentrations in wine samples from the Taman zone, (µg/L).

Elements	Subset 1		Subset 2		Subset 3		Subset 4		Subset 5	
	Left Bound	Right Bound	Left Bound	Right Bound	Left Bound	Right Bound	Left Bound	Right Bound	Left Bound	Right Bound
Al	488	1485	239	1734	0	1983	0	2232	0	2481
Ba	81	191	54	218	26	246	0	273	0	301
Ca	50,043	81,296	42,230	89,110	34,417	96,923	26,604	104,736	18,790	112,549
Cu	0	778	0	1082	0	1385	0	1688	0	1992
Fe	1648	5044	799	5893	0	6742	0	7591	0	8440
K	498,290	973,612	379,459	1,092,443	260,629	1,211,273	141,798	1,330,104	22,968	1,448,935
Li	16	40	10	46	4	52	0	59	0	65
Mg	72,844	167,334	49,222	190,956	25,600	214,579	1977	238,201	0	261,823
Mn	835	1910	566	2179	297	2448	28	2717	0	2986
Na	25,377	75,654	12,807	88,223	238	100,793	0	113,362	0	125,931
Ni	0	436	0	603	0	771	0	938	0	1106
Rb	0	4252	0	5357	0	6462	0	7567	0	8672
Sr	677	1426	489	1613	302	1801	115	1988	0	2175
Ti	3	25	0	30	0	36	0	41	0	47
Zn	0	1324	0	1688	0	2051	0	2415	0	2778

**Table 3:** Bounds of data on element concentrations in wine samples from the Black Sea zone, (µg/L).

Elements	Subset 1		Subset 2		Subset 3		Subset 4		Subset 5	
	Left Bound	Right Bound	Left Bound	Right Bound	Left Bound	Right Bound	Left Bound	Right Bound	Left Bound	Right Bound
Al	30	3132	0	3908	0	4683	0	5459	0	6234
Ba	76	169	52	192	29	216	6	239	0	262
Ca	54,611	78,465	48,648	84,428	42,684	90,392	36,721	96,355	30,758	102,319
Cu	0	457	0	586	0	714	0	843	0	971
Fe	1681	5793	653	6821	0	7849	0	8877	0	9905
K	457,912	1,149,807	284,938	1,322,781	111,964	1,495,755	0	1,668,729	0	1,841,703
Li	6	39	0	48	0	56	0	65	0	73
Mg	53,047	102,803	40,608	115,242	28,169	127,680	15,730	140,119	3291	152,558
Mn	829	1517	657	1689	485	1861	313	2034	141	2206
Na	19,688	72,485	6489	85,684	0	98,884	0	112,083	0	125,282
Ni	7	83	0	102	0	121	0	140	0	159
Rb	468	1149	298	1319	128	1489	0	1659	0	1829
Sr	639	1614	396	1858	152	2102	0	2345	0	2589
Ti	5	27	0	33	0	38	0	44	0	49
Zn	420	910	297	1033	175	1155	52	1278	0	1400

**Table 4:** Bounds of data on element concentrations in wine samples from the South foothill zone, (µg/L).

Elements	Subset 1		Subset 2		Subset 3		Subset 4		Subset 5	
	Left Bound	Right Bound	Left Bound	Right Bound	Left Bound	Right Bound	Left Bound	Right Bound	Left Bound	Right Bound
Al	597	1678	326	1948	56	2219	0	2489	0	2759
Ba	90	273	45	318	0	364	0	409	0	455
Ca	48,289	78,707	40,685	86,311	33,081	93,915	25,477	101,519	17,872	109,124
Cu	26	86	12	101	0	116	0	130	0	145
Fe	2544	5110	1902	5752	1261	6393	619	7035	0	7677
K	626,346	1,292,538	459,798	1,459,086	293,250	1,625,634	126,702	1,792,182	0	1,958,731
Li	8	21	4	24	1	28	0	31	0	34
Mg	61,470	122,086	46,316	137,240	31,162	152,394	16,008	167,549	854	182,703
Mn	579	1624	317	1886	56	2147	0	2409	0	2670
Na	19,674	63,055	8828	73,901	0	84,746	0	95,592	0	106,437
Ni	0	154	0	203	0	252	0	301	0	350
Rb	91	1988	0	2463	0	2937	0	3411	0	3886
Sr	468	1425	228	1665	0	1904	0	2144	0	2383
Ti	10	39	3	46	0	53	0	61	0	68
Zn	402	825	297	930	191	1036	86	1142	0	1247

**Table 5:** Quantitative distribution of wine samples by zones and subsets.

Subset	Zone			Total
	Taman	Black Sea	South Foothill	
1	8	5	7	20
2	80	26	17	123
3	122	44	42	208
4	146	60	50	256
5	177	64	65	306
6	210	77	72	359
7	210	77	72	359

**Table 6:** Clustering metrics for subsets 1–7.

Metric	Subset 1	Subset 2	Subset 3	Subset 4	Subset 5	Subset 6	Subset 7
S-S	0.58	0.53	0.35	0.35	0.27	0.26	0.03
C-H	0.93	0.59	0.05	0.02	0.06	0.07	0.04
D-B	1.79	3.68	8.62	21.19	18.97	16.87	13.16
Dunn	0.44	0.05	0.02	0.01	0.007	0.007	0.0005

**Table 7:** The results of building neural network models for subsets 1–7.

Subset	Network Name	Training Performance	Test Performance	Training Algorithm	Error Function	Hidden Activation	Output Activation
1	MLP 15-15-3	100	100	BFGS 5	Entropy	Logistic	Softmax
2	MLP 15-11-3	100	100	BFGS 7	Entropy	Identity	Softmax
3	MLP 15-12-3	100	98.08	BFGS 15	Entropy	Hyperbolic	Softmax
4	MLP 15-15-3	100	97.37	BFGS 15	Entropy	Logistic	Softmax
5	MLP 15-9-3	99.13	97.37	BFGS 76	Sum of Squares	Hyperbolic	Identity
6	MLP 15-15-3	100	93.25	BFGS 29	Entropy	Hyperbolic	Softmax
7	MLP 15-15-3	91.85	3.37	BFGS 55	Entropy	Hyperbolic	Softmax

**Table 8:** Results of building neural network models for each of the 10 stages.

№ Stage	Dataset Size	Network Name	Training Performance	Test Performance	Training Algorithm	Error Function	Hidden Activation	Output Activation
1	36	MLP 15-5-3	100	100	BFGS 10	SOS	Identity	Logistic
2	72	MLP 15-15-3	100	100	BFGS 11	Entropy	Logistic	Softmax
3	108	MLP 15-15-3	100	100	BFGS 12	Entropy	Logistic	Softmax
4	144	MLP 15-12-3	100	100	BFGS 15	Entropy	Tanh	Softmax
5	180	MLP 15-12-3	100	98.19	BFGS 16	Entropy	Tanh	Softmax
6	216	MLP 15-16-3	100	95.31	BFGS 20	Entropy	Logistic	Softmax
7	252	MLP 15-12-3	100	93.33	BFGS 24	Entropy	Tanh	Softmax
8	288	MLP 15-15-3	98.51	97.67	BFGS 114	SOS	Exponential	Exponential
9	324	MLP 15-15-3	100	93.81	BFGS 180	SOS	Exponential	Exponential
10	359	MLP 15-9-3	100	93.25	BFGS 106	SOS	Tanh	Identity

**Table 9:** The results of building neural network models for 10 stages.

Metrics	Dataset Sizes									
	36	72	108	144	180	216	252	288	324	359
S-S	0.45	0.49	0.50	0.37	0.35	0.32	0.30	0.26	0.26	0.26
C-H	0.09	0.41	0.37	0.31	0.19	0.09	0.11	0.07	0.07	0.07
D-B	7.15	3.62	14.93	5.74	3.36	11.46	15.19	15.63	17.16	16.87
Dunn	0.17	0.12	0.04	0.03	0.02	0.02	0.01	0.007	0.007	0.007

## Data Availability

The original contributions presented in this study are included in the article and the Supplementary Materials. Further inquiries can be directed to the corresponding author.

## Supplementary Materials

The following supporting information can be downloaded at: https://www.mdpi.com/article/10.3390/foods15142511/s1, Table S1: Data on the main characteristics of wines.
